# Simultaneous acromioclavicular dislocation, proximal humeral fracture, and reverse Hill–Sachs lesion: a case report

**DOI:** 10.1186/s13256-023-03966-2

**Published:** 2023-05-30

**Authors:** Hasan Barati, Sina Afzal

**Affiliations:** Department of Orthopedic, School of Medicine, Imam Hossein Hospital, Shahid Beheshti University of Medical Sciences, Tehran, Iran

**Keywords:** Arthroscopy, Acromioclavicular dislocation, Proximal humeral fracture, Reverse Hill–Sachs lesion, Case report

## Abstract

**Background:**

In cases with injuries to the shoulder region, the combination of acromioclavicular joint dislocation, reverse Hill–Sachs lesion, and proximal humeral fracture is a very rare condition.

**Case presentation:**

This study described a 38-year-old male Persian patient with simultaneous acromioclavicular joint dislocation, proximal humeral fracture, and reverse Hill–Sachs lesion due to motor vehicle crash injury who underwent arthroscopic acromioclavicular joint fixation using tight rope technique. In the 7-month follow-up period following the surgical fixation, range of motion was approximately normal. Reduction and hardware were intact, no dislocation or apprehension to dislocation was observed. Patient only had minor shoulder pain at the end of range of motion and a dull pain on the site of incision over the clavicle in deep touch. Our findings showed acceptable arthroscopic outcomes in the management of such complex case.

**Conclusion:**

Our experience on this case showed acceptable outcomes of the arthroscopic treatment of the acromioclavicular joint dislocation in the management of such a complex case with associated injuries to the shoulder region.

## Introduction

Acromioclavicular joint (ACJ) dislocations account for about 12% of all shoulder girdle injuries [1]. Although evidence shows proximal humeral fracture (PHF) and ACJ injuries having incidence rates of 6% and 9%, respectively [2], the mechanisms of fractures in these two injuries are different, making the coincidence of these two injuries unlikely. Concomitant shoulder girdle injuries are common with high-grade ACJ dislocation and posterior shoulder dislocation [3, 4]. However, the combination of ACJ dislocation and reverse Hill–Sachs lesion and PHF is a very rare condition. Timely diagnosis of these simultaneous injuries without missing anything and providing an appropriate treatment approach are challenging and confusing for orthopedic surgeons. In this study we report a rare case with simultaneous acromioclavicular joint dislocation, proximal humeral fracture, and reverse Hill–Sachs lesion due to motor vehicle crash injury, managed by arthroscopic surgery, and review the surgical approach and outcomes of surgery for the follow-up period.

## Case presentation

**Fig. 1:**
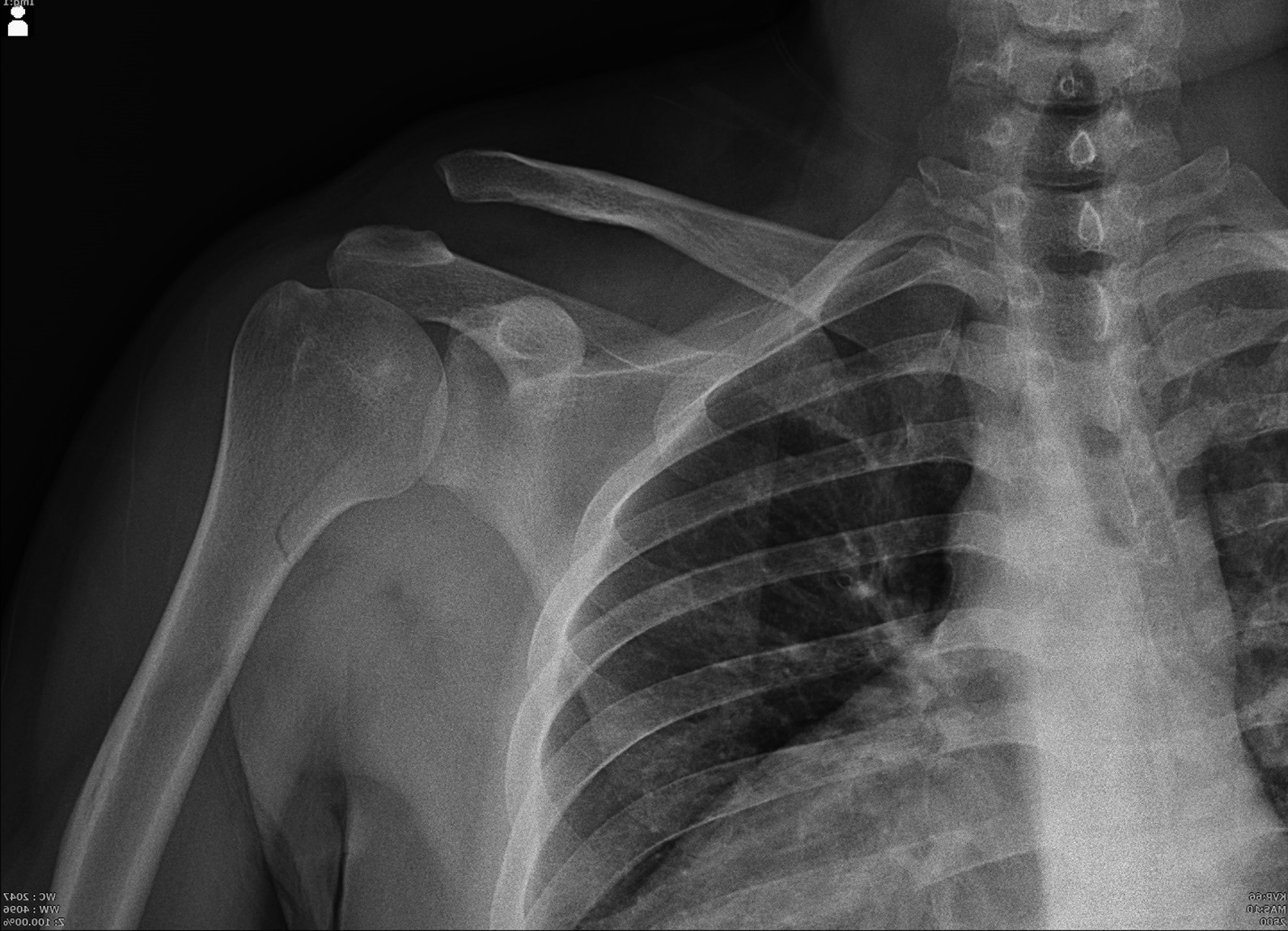
Concomitant Rockwood type V ACJ dislocation and a non-displaced unicortical proximal humerus fracture in digital X-ray

**Fig. 2:**
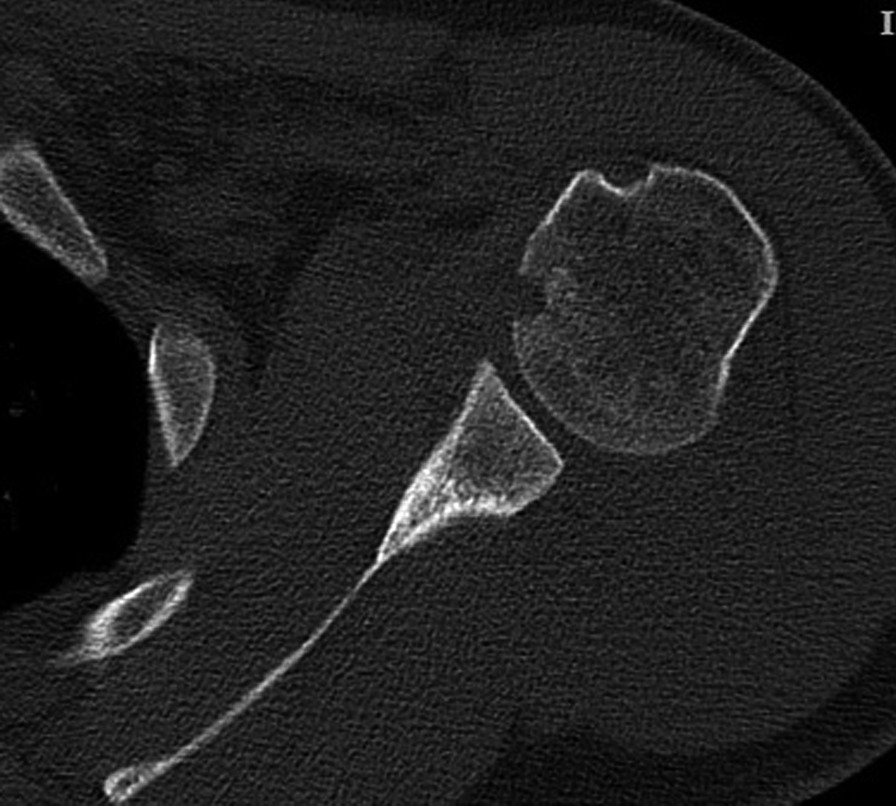
Small impacted fracture in the anteromedial of the humeral head indicative of the reverse Hill–Sachs lesion in three-dimensional computed tomography (3D CT) scan

A 38-year-old right-handed male Persian patient was referred to a tertiary medical center due to a motor vehicle crash injury. He was an auto mechanic with no significant past medical history. His digital X-ray showed concomitant Rockwood type V ACJ dislocation and a non-displaced unicortical PHF (Fig. 1). There were no further injuries elsewhere. On physical examination, deformity, ecchymosis, severe tenderness, and swelling at the top of the right shoulder were found. Instability tests were not performed due to severe shoulder pain. Neurovascular examinations and all laboratory values were unremarkable. A three-dimensional computed tomography (3D CT) scan was conducted that revealed a small impacted fracture in the anteromedial humeral head indicative of reverse Hill–Sachs lesion (Fig. 2). Regarding the associated soft tissue and labrum injury, since the patient was a candidate for arthroscopic-assisted evaluation and reconstruction of the ACJ injury, further imaging evaluation such as magnetic resonance imaging was waived before surgery, since the arthroscopy provides more accuracy and details of the joint and accompanying injuries.

### Surgical technique

The operation initiated with beach chair positioning of the patient under general anesthesia. Glenohumeral (GH) instability tests (Jerk and Kim tests) were done before the surgical site preparation and were negative. Following the sterilization of surgical field, standard posterior portal of shoulder arthroscopy was performed. The diagnostic arthroscopy revealed a reverse Hill–Sachs lesion with less than 10% involvement of the humerus head and less than 2 mm in depth. Unexpectedly, the posterior labrum was intact on arthroscopic evaluation. Other GH joint structures were normal, except for the biceps tenosynovitis.

A 2 cm incision was then made 3–4 cm medial to ACJ for placing the upper part of jig. Following the anterior portal establishment, subcoracoid release was performed by shaver and electrocautery. Accordingly, full visualization of the coracoid base was attained and lower part of jig was placed under it. Upper part of the jig was placed above the clavicle. Guide pin was placed in an appropriate location, using jig device. Both the clavicle and coracoid were drilled via the 4-mm-canulated reamer.

Following the extraction of the guide pin, the tightrope passed through the tunnel by a flexible wire. The Endobutton was passed through the tunnel in a longways manner and after placing under the coracoid perpendicularly, the AC joint reduction was applied and then tight rope was tightened over the clavicle. Reduction was obtained by applying upward pressure under the elbow and downward over the clavicle. As the adequate reduction under direct arthroscopically vision of coracoclavicular interval was achieved, the tightrope was tightened. Reduction was checked through the use of the intraoperative Zanka view, and double checked by fluoroscopy thereafter. Due to the negative posterior shoulder instability tests and the involvement of the humeral head less than 20%, the reverse Hill–Sachs lesion did not require surgical treatment in this patient. The extremity was supported with sling and swathe. The patient was discharged the next day.

### Follow-up recommendations and protocols

Patient was advised to rest his arm in a sling and swathe for 4 weeks for pain alleviation and gravitational forces control. Afterward, passive exercises, including shoulder pendulum exercises, were begun for 2 weeks. As the last step of postoperation care, ten sessions of physiotherapy were done to regain his normal range of motion (ROM), and ten more sessions for strengthening of his muscles. Returning to sport activities was restricted for 5 months after the surgery.

### Follow-up outcomes

**Fig. 3:**
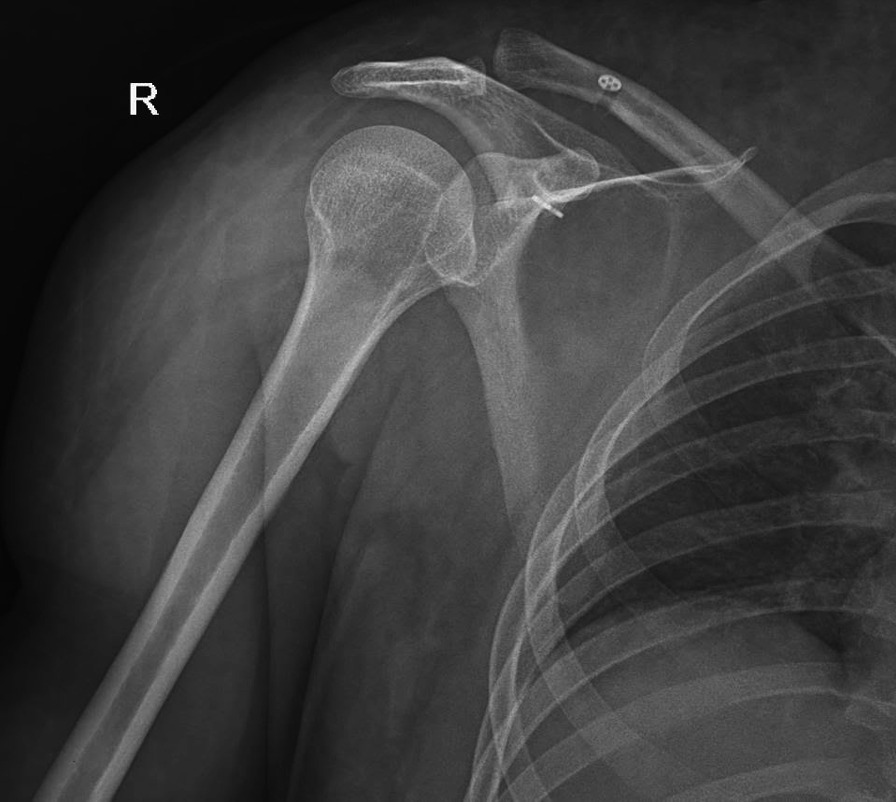
Imaging of shoulder in the 7-month follow-up after arthroscopic management of the reported case

In the 7-month follow-up period following the surgical fixation, ROM was approximately normal. Reduction and hardware were intact and no dislocation or apprehension to dislocation were reported (Fig. 3). A minor shoulder pain at the end of ROM and a dull pain on the site of incision over the clavicle in deep touch were detected. No sign of infection was found during the follow-up period.

## Discussion

The goal of treatment of ACJ injuries is to maintain the shoulder kinematics, and this is attainable by restoring the anatomy of the joint. For this purpose, several methods have been introduced to date, such as coracoclavicular screw, hook plate, and tightrope [5]. Approximately 30% of ACJ injuries have a simultaneous injury in GH joint [6]. As all these injuries are not bony, a comprehensive treatment strategy and evaluation are required before ACJ fixation.

To the best of our knowledge, no similar study was reported previously. In 2020, Tofts *et al*. reported a patient with posterior shoulder dislocation, posterior labrum damage, reverse Hill–Sachs lesion concomitant with Rockwood type V, and ACJ dislocation [7]. In contrast with that study, our reported patient had proximal metaphyseal humeral fracture and intact posterior labrum. As the intact labrum was detected in our patient, the relocated posterior shoulder dislocation was improbable. They described same arthroscopic surgical technique with acceptable outcomes [7], and in line with their report, excellent outcomes were shown in our patient managed by a similar surgical technique. In 2013, Pauly *et al*. reported a case series of 125 patients with high-grade ACJ injuries operated with arthroscopic surgical techniques and concluded that arthroscopic approach provides optimal surgical results [6]. As mentioned before, posterior shoulder dislocation was not seen in the current case, which makes this report unique.

Compared with chronic ACJ dislocation, acute ACJ injury could be much more challenging and problematic due to the associated injuries and obstacles in diagnostic evaluations such as MRI, which is likely to have poor quality due to motion artifacts caused by patient pain and more likely to occur in acute cases of ACJ injuries than in chronic cases [8]. In the current reported case, clinical examinations were difficult due to severe shoulder pain, and the results of imaging modalities were suboptimal in quality due to possible artifacts, as stabilizing the patient for imaging was difficult. Our medical center is a tertiary referral center; all the instruments that may be needed for GH joint pathologies are available. We then decided to fix the ACJ arthroscopically to see the whole GH joint by direct vision and treat all the patient’s injuries in one stage to lower the costs imposed to both the healthcare system and the patient. The acceptable outcome of the arthroscopic approach used to manage the injuries in the reported case make this approach a viable option in patients with complex injuries in the shoulder region.

## Conclusion

Concomitant GH injuries such as PHF and reverse Hill–Sachs lesion should be noted in the ACJ injuries. On the basis of the results of the reported case, we recommend arthroscopic approach as the treatment strategy to detect all GH pathologies and treat them in one stage in such cases.

## Data Availability

The material presented in this study are available from the corresponding author on reasonable request.

## Abbreviations

ACJ: Acromioclavicular joint
PHF: Proximal humeral fracture
3D CT: Three-dimensional computed tomography
GH: Glenohumeral
ROM: Range of motion
